# From neighborhoods to brain health: an outreach model linking health literacy, cognitive care, research, and the arts, in San Francisco

**DOI:** 10.3389/fpubh.2026.1930344

**Published:** 2026-09-17

**Authors:** Serggio Lanata, Charles Windon, Boon Lead Tee, Jessica de Leon, Gloria Annette Aguirre, Ashley J. Jackson, Kevin Lieu, Valentina Diaz, Stefanie Pina Escudero, Tanisha G. Hill-Jarrett, Andrea Weng, Kunle Adewale, Alinda Amiuri, Jorge Archila, Laura Maria Calderon, Alyssa Chen, Yu Chen, Annie Ching, Aaron Colverson, Catherine Correa Lopera, Karen Dorsman, Berenice Fuentes-Juarez, Araceli Garcia, Ariel Guevarra, Yoon Mee Joo, Magda Kaczmarska, Kiggundu R. Erismas, Jana Klein, Alex Kornhuber, Susana Lay, Fangda Leng, Sophia W. Light, Kevin Li, Lucia Lopez, Daniel Macri, Eugenie Mamuyac, Luis Martinez Roman, Diana Mei, Maritza Pintado Caipa, Caroline Prioleau, Rochelle-Jan Dionisio Reyes, Oscar Robles-Archila, Diana Rodriguez, Ida Shibiru, John Matthew Suntay, Kamryn Thomas, Miwa Tucker, Yun-Ting Wang, Joanne Wu, XingGe Zhao, Karen Smith, Maria Luisa Gorno-Tempini, William W. Seeley, Howard J. Rosen, Victor Valcour, Joel H. Kramer, Katherine Possin, Gil Rabinovici, Bruce L. Miller

**Affiliations:** 1 Edward and Pearl Fein Memory and Aging Center, Department of Neurology, Weill Institute for Neurosciences, University of California, San Francisco, San Francisco, CA, United States; 2 Global Brain Health Institute (GBHI), University of California, San Francisco, San Francisco, CA, United States; 3 Departments of Psychiatry and Pediatrics, University of Texas Southwestern Medical Center, Dallas, TX, United States; 4 Department of Psychiatry & Behavioral Neuroscience, The University of Chicago, Chicago, IL, United States; 5 School of Nursing, University of Pennsylvania, Philadelphia, PA, United States; 6 Geisel School of Medicine, Dartmouth College, Hanover, NH, United States; 7 Department of Psychology, California State University Long Beach, Long Beach, CA, United States

**Keywords:** ADRC, Alzheimer’s disease, brain health, community outreach and engagement, dementia, neighborhood health disparities, neurocognitive disorders in aging, social and environmental determinants of health

## Abstract

Traditional community outreach, research recruitment, and engagement strategies in aging neuroscience often fail to target and capture the diversity of structural and environmental conditions that shape the risk of age-related neurocognitive disorders (NCDs), including Alzheimer’s disease. In this manuscript, we describe the rationale, implementation, and outcomes of the Community Outreach Program (COP) of the UCSF Fein Memory and Aging Center (MAC), emphasizing its neighborhood-based initiative designed to engage vulnerable populations to improve brain health literacy, access to cognitive care, and research inclusion among older adults living in underserved San Francisco neighborhoods. Guided by a social and environmental determinants of brain health framework, the program used neighborhood-level vulnerability and research representation mapping to identify neighborhoods with high vulnerability and low engagement in research, then implemented a multilingual, community-engaged model centered on four pillars: brain health education, arts- and movement-based programming, community-based cognitive clinics, and multilingual media outreach. Delivered in partnership with community organizations and safety-net providers in Mandarin, Cantonese, Spanish, Tagalog, and English, this model has generated durable partnerships across vulnerable neighborhoods, expanded access to culturally responsive cognitive care, improved inclusion in brain aging and NCDs research, and fostered innovative research projects. From 2019 to 2025, representation of individuals from vulnerable and underrepresented San Francisco neighborhoods in UCSF Fein MAC observational research increased substantially compared to previous years. These findings support place-based, multilingual, and reciprocity-centered community engagement as a promising framework for reducing and studying disparities in brain health.

## 1 Introduction

According to the United States (U. S.) Census, by 2030, all baby boomers will be 65 or older, and by 2034, older adults will outnumber children for the first time in U. S. history (1). This unprecedented demographic transformation poses serious public health challenges, foremost among them the rise of age-related neurocognitive disorders (NCDs) such as Alzheimer’s disease (AD). Neurological disorders, in particular age-related NCDs, are now a leading cause of disability and ill health worldwide, accounting for a substantial proportion of disability-adjusted life years (2). The burden of these disorders varies across populations and communities, largely due to disparities in exposure to potentially modifiable factors such as socioeconomic conditions, access to health care, education, health literacy, and other structural determinants of late-life brain health.

All forms of NCDs lead to progressive cognitive decline that begins with prodromal or mild stages and culminates in a clinical syndrome known as dementia, defined as cognitive impairment severe enough to impede independent living. Alzheimer’s disease, the most common NCD and therefore the leading cause of dementia globally, affects an estimated 7.2 million Americans aged 65 and older - a figure projected to nearly double by 2060 (3). The diagnosis and management of NCDs remain challenging due to their long and slowly evolving course, poor recognition of early signs and symptoms, long illness duration, and the high costs of care. Many individuals remain undiagnosed for years, particularly when presenting with non-memory symptoms such as language or behavioral dysfunction (4, 5). These patterns contribute to excessive healthcare utilization, increased spending, diminished quality of life, and racial and ethnic disparities (6). In the U. S. alone, the total annual cost of dementia care in 2025 reached $384 billion (3), with projected costs of formal and informal care expected to reach trillions of dollars by 2060 (7).

Encouragingly, recent advances in prevention, diagnosis, and treatment of NCDs offer new opportunities to alter this trajectory. Research suggests that up to 45% of NCDs may be preventable through public health interventions targeting modifiable medical, behavioral, and environmental factors (8). The field has entered a new era of biological diagnostic and therapeutic precision: novel minimally invasive biomarker tests (9), especially blood-based assays, allow for earlier and more accurate diagnosis of AD and other NCDs years before dementia stages of illness, while newly approved disease-modifying therapies have introduced the first pharmacologic options capable of slowing disease progression (10). However, these advances will only achieve their full potential if early recognition and access to diagnosis and care improve, particularly in primary care settings and among medically underserved, socioeconomically vulnerable populations.

Academic neuroscience centers play a unique and powerful role in improving population awareness of early neurocognitive decline, strengthening referral pathways to research, and facilitating access to emerging diagnostics and therapies. In the U. S., many of these efforts occur through the National Institute on Aging–funded Alzheimer’s Disease Research Center (ADRC) programs, a nationwide network of academic centers dedicated to research on age-related NCDs. Within this network, ADRC Outreach, Recruitment, and Engagement (ORE) Cores help disseminate information about brain health to local communities, build community partnerships, and ensure the creation and maintenance of research cohorts representative of the U. S. population. At the University of California, San Francisco (UCSF) Fein Memory and Aging Center (MAC), the Community Outreach Program (COP) emerged from the ADRC ORE Core, supported by the UCSF Global Brain Health Institute (GBHI). Over the years, the COP expanded with support of other NIH-funded research projects to sustain community engagement and research participation across different Fein MAC research projects beyond the ADRC, further engaging San Francisco’s diverse aging communities.

The Fein MAC COP employs a multimodal outreach and recruitment strategy that includes engagement approaches focused on race and ethnicity, primary language, specific NCDs, and other factors. In parallel, over the past few years, we have developed a neighborhood-based approach that uses geographically defined communities as the primary unit of engagement. This manuscript focuses on this neighborhood-based framework, examining its conceptual rationale in the context of San Francisco’s complex history of migration and present-day sociodemographic heterogeneity, and detailing how such an approach can be operationalized through community-based partnerships, locally tailored programming, and iterative collaborations. We describe ongoing efforts to evaluate its impact, including metrics of reach, participation, and downstream enrollment at the UCSF ADRC. While this neighborhood-based approach is particularly well-suited to highly sociodemographically diverse urban settings, we argue that it may also be relevant in regions that are relatively homogeneous in racial or ethnic composition but nonetheless exhibit meaningful disparities in brain health driven by structural and environmental factors.

## 2 The San Francisco context

### 2.1 Brain health challenges in California and San Francisco

The challenges and opportunities associated with the rise of age-related NCDs are especially pressing in California, where approximately 660,000 adults aged 65 and older were living with AD in 2019 (11). This number is projected to more than double by 2040, far outpacing general population growth. Other causes of NCDs, including cerebrovascular disease and Lewy body disease, each accounting for 5–10% of statewide cases, are also expected to increase exponentially (11). The economic impact is equally concerning. Dementia-related care costs in California alone, estimated at $67 billion in 2019, are projected to exceed $150 billion by 2040 (11). This steep increase underscores the urgency for prevention-oriented public health strategies that emphasize education, early detection and diagnosis, and coordinated care. Racial and ethnic minority groups shoulder a disproportionate share of the burden, with the number of Californians with suspected AD projected to more than triple among Latino Americans, nearly triple among Black/African Americans, and more than double among Asian American and Pacific Islander populations between 2019 and 2040 (11). These brain health disparities are rooted in modifiable factors, not in immutable and heritable racial or ethnic factors (12).

The City and County of San Francisco recognized the scope of these challenges in its 2009 Dementia Strategic Plan (13, 14), which called for improved brain health education for its diverse communities and primary care providers, as well as expanded capacity for early diagnosis, integrated care, and prevention to mitigate these disparities. The UCSF ADRC is uniquely positioned to drive sustained progress to meet these challenges because it is embedded within the UCSF Fein MAC ecosystem of cutting-edge neuroscience and translational research on age-related NCDs, allowing it to draw directly on the latest scientific advances and institutional resources while feeding community-identified priorities back into research planning and implementation.

### 2.2 The rich sociodemographic composition of San Francisco

As a historic port city, San Francisco has long served as a destination for global migration, particularly during the 19th-century Gold Rush, resulting in intersecting layers of cultural and socioeconomic diversity that defy conceptual and operational classifications based on standard U. S. Census racial and ethnic categories.

Nearly 40% of the city’s 810,000 residents identify as non-Latino White, yet this group encompasses a wide range of ethnic ancestries, including Irish, Italian, German, Russian, Eastern European, and Middle Eastern heritage, along with more recent arrivals from across the U. S. Asian Americans, who make up roughly one-third of the population, are predominantly diverse Chinese Americans but also include substantial Filipino, Indian, Vietnamese, Korean, and Japanese communities, each with a multitude of distinct linguistic and cultural traditions that meaningfully and differentially shape health behaviors and health outcomes. Similarly, Latino residents, comprising about one-sixth of the population, trace roots to Mexico, Central and South America, and the Caribbean, with many families further reflecting diverse Indigenous, African, and European influences. Likewise, Black San Franciscans, now representing about 5% of residents, come from all corners of the U. S. and embody a rich tapestry of customs and traditions that resist homogenizing categories.

When viewed over two centuries of immigration, San Francisco’s complex demographics extend well beyond racial and ethnic boundaries to include many other significant dimensions that play a direct role in shaping health, such as English proficiency, acculturation, and economic affluence, among others. For example, approximately 43% of San Francisco residents over the age of five speak a language other than English at home; accordingly, municipal agencies offer adult services in more than 40 languages, a reality that greatly challenges equitable health care delivery in the city (15). Socioeconomically, a 2023 needs assessment for San Francisco calculated that a family of four requires $164,713 per year to cover basic expenses, including housing, childcare, food, and transportation; this is more than five times the federal poverty level (16). About half of families in San Francisco earn below this self-sufficiency threshold, and this economic vulnerability affects all racial and ethnic groups, albeit differently. According to the Bay Area Equity Atlas (a comprehensive data support system incorporating U. S. Census and other demographic data), among San Francisco Bay Area residents in the very low-income group - households whose income is less than 50% of the Area Median Family Income - approximately 35% identify as Latino, 25% as Asian, 26% as White, and 10% as Black (17).

The practical implications of San Francisco’s socioeconomic and demographic complexity for brain health outreach are far-reaching. Efforts to promote cognitive health and study brain health disparities in San Francisco therefore must move beyond targeting only U. S. Census-defined racial or ethnic categories and engage with the grounded realities of language proficiency, local cultural/ethnic practices, and other environmental and economic factors that differentially drive brain health outcomes across racial and ethnic groups in the city (18, 19).

## 3 The community outreach program (COP) of the UCSF Fein MAC

### 3.1 Recognizing local social and environmental factors as the main drivers of brain health disparities in San Francisco

The intertwining of historical, cultural, linguistic, economic, and other health-influencing factors in San Francisco has produced richly diverse communities that are arguably best understood at the neighborhood level. The Bayview - Hunters Point neighborhood, for example, is one of the most racially and ethnically diverse neighborhoods in the city (approximately 35% Asian American, 28% Black/African American, 22% Latino American, and 15% White), and it exemplifies how local environmental exposures and economic deprivation interact to shape the health of its residents (20). This neighborhood is affected by long history of air pollution (21), an independent risk factor for age-related NCDs (22). Moreover, the neighborhood has double the citywide unemployment rate, more than double the poverty rate, and one of the lowest high school graduation rates in the city (23). Chronic exposure to such environmental and economic adversity creates cumulative physiological stress, sometimes described as allostatic load, that undermines the health of all residents in this neighborhood, although not all racial and ethnic groups are affected equally (24).

Conversely, the contrasting health profiles of the Chinatown and Richmond neighborhoods further illustrate the impact of local environments on health. Although both communities are home to large Chinese American populations, their health outcomes differ substantially, largely due to neighborhood-level variations in social, structural, environmental, and economic conditions (25). Chinatown is characterized by high population density, limited green spaces, older housing, and lower household incomes, which contribute to increased risks of chronic diseases associated with crowding, pollution, and economic stress (26). By contrast, the Richmond District benefits from greater access to parks, higher household incomes, better air quality, and newer housing. These neighborhood profiles demonstrate that the same racial or ethnic identification does not necessarily confer a similar health risk profile; rather, neighborhood environments, local resources, and other structural conditions are often stronger determinants of population health (27).

Indeed, each San Francisco neighborhood embodies distinct structural, social, and environmental determinants of health, resulting in different community health profiles that defy race- or ethnicity-based explanations (28). Aggregated racial and ethnic categorizations overlook the neighborhood-level structural and environmental forces that shape residents’ daily lives and health. Consequently, any effort to understand brain health disparities in San Francisco or to design effective interventions must engage with the complex and fluid lived realities of residents and look beyond treating race and ethnicity as static, independent predictors of brain health risk.

### 3.2 Emphasizing neighborhood health

The Fein MAC COP has embraced a place-based, multilingual, multicultural approach that prioritizes the lived experiences of San Francisco residents at a neighborhood level, in alignment with the San Francisco Department of Public Health framework (29). This approach recognizes that differences in brain health are ultimately shaped by potentially modifiable upstream social and environmental conditions (Figure 1) (8, 30). Within this neighborhood-based framework, race and ethnicity are operationalized as fluid markers of social risk, not as categorical predictors of brain health. This perspective also aligns with socioecological models, which emphasize the multilevel pathways through which neighborhood context and cumulative stressors shape health trajectories over the life course (24, 27, 31).

**Figure 1:**
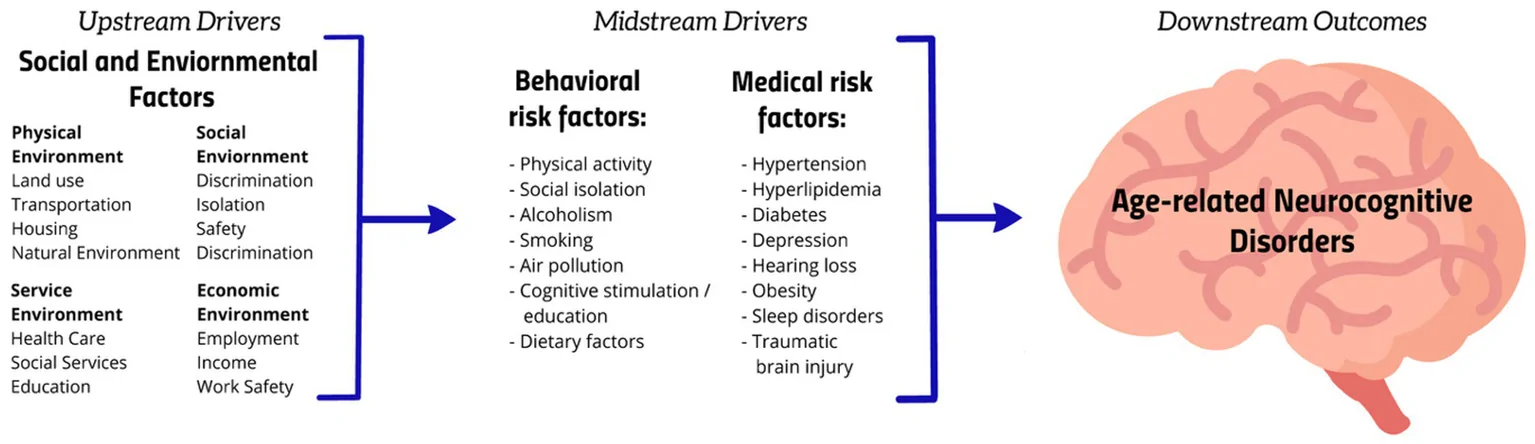
Midstream and upstream factors driving downstream neurocognitive disorders. Epidemiological evidence shows that disparities in the incidence and prevalence of age-related neurocognitive disorders are driven primarily by midstream behavioral/lifestyle and medical risk factors, which in turn are influenced by more upstream social and environmental factors that are potentially modifiable. The work of the Fein MAC COP is guided by this evidence-based framework of brain health disparities (7, 8).

Accordingly, in 2018, we used neighborhood-level data collected by the San Francisco Department of Public Health on social and environmental determinants of health to generate a San Francisco neighborhood vulnerability map (Figure 2), which informed us of which neighborhoods were the most and the least vulnerable. We then produced a complementary map (Figure 3) showing the neighborhood-level representation of individuals who had enrolled in the UCSF ADRC up to 2018, prior to the launch of the Fein MAC COP. An overlapping analysis of these maps demonstrated that as of 2018 the most vulnerable neighborhoods in San Francisco were among the most underrepresented in the UCSF ADRC, and these vulnerable and underrepresented neighborhoods are also the neighborhoods most densely populated by historically marginalized racial and ethnic groups - especially Asian-Americans, Black/African Americans, and Latino Americans - reflecting the long and complex histories of these groups in San Francisco, as discussed above.

**Figure 2:**
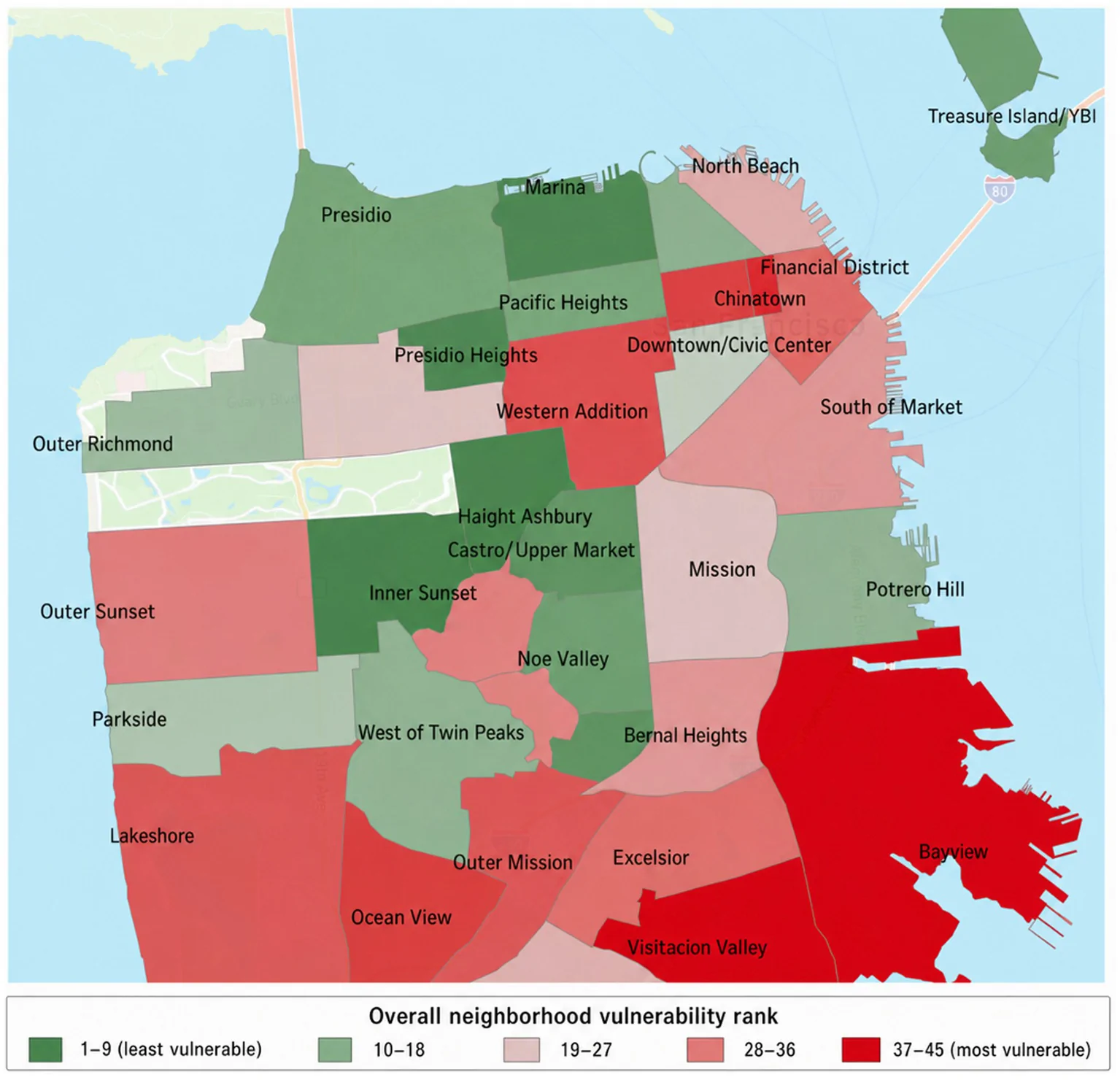
Neighborhood vulnerability rankings across San Francisco. Map of San Francisco neighborhoods classified according to their overall vulnerability rank. Neighborhood vulnerability scores were calculated using a composite ranking method that integrated fourteen key indicators of social, economic, environmental, and safety factors collected by the San Francisco Department of Public Health. Neighborhood indicators associated with greater vulnerability (e.g., low-income households, rent burden, evictions, crime, high-injury corridors, etc.) were ranked and summed to produce a negative vulnerability score. Indicators representing protective neighborhood resources (e.g., pedestrian environment, tree canopy, proximity to community centers, residential mobility, food access, etc.) were similarly ranked and combined into a positive resource score. These component ranks were summed to generate an overall relative vulnerability ranking across neighborhoods, ranging from 1 (least vulnerable) to 45 (most vulnerable). Neighborhoods are displayed in five color categories: Dark green, ranks 1–9; light green, ranks 10–18; light gray, ranks 19–27; salmon, ranks 28–36; and dark red, ranks 37–45. Thus, progressively redder colors indicate greater relative neighborhood vulnerability. The same overall vulnerability ranks are reported for the corresponding neighborhoods in Table 1.

**Figure 3:**
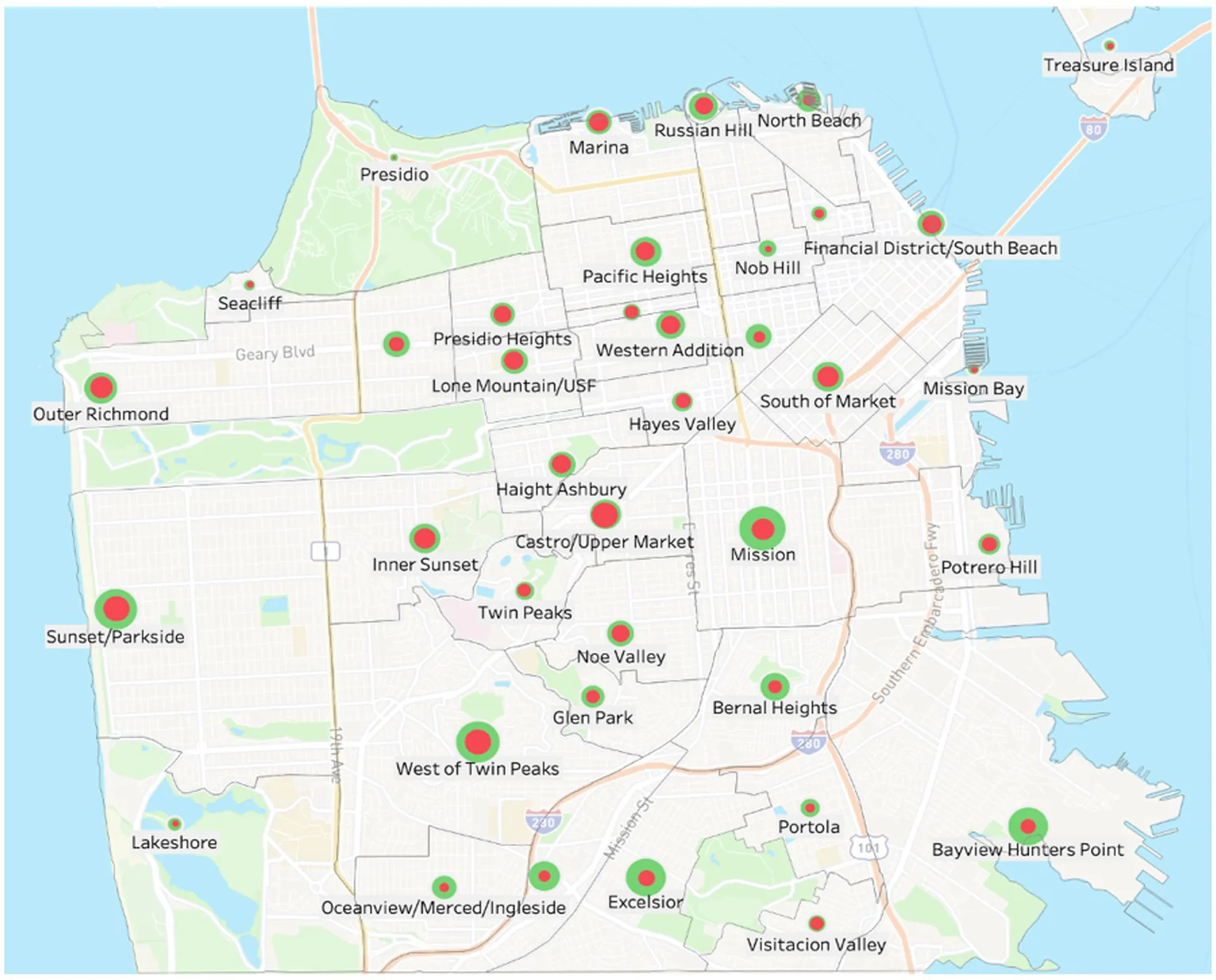
UCSF ADRC new participant representation across San Francisco neighborhoods from 2010 to 2025. Red circles denote 2010–2018 participant representation per neighborhood, and green circles denote 2019–2025 participant representation per neighborhood. The size of circles within each neighborhood indicates the number of newly enrolled ADRC research participants residing in that neighborhood, with circle size proportional to participation count. Increased circle size and broader representation of vulnerable and underrepresented neighborhoods demonstrate the effects neighborhood-based expansion of the UCSF Fein MAC COP activities.

This knowledge informed our program’s neighborhood-based outreach and engagement approach and helped us operationally define neighborhoods that were vulnerable and underserved and underrepresented to the UCSF Fein MAC.

### 3.3 Neighborhood-based approach

Over the past eight years, we have established relationships with 65 partners in San Francisco (Supplementary Table 1). Some of these relationships are short-lived; others have lasted more than 8 years, and most of these partners are in highly vulnerable neighborhoods in the city, which are also underrepresented in the UCSF ADRC. In collaboration with these partners, our approach integrates community-based participatory research and advisory boards, multilingual engagement, clinical integration, and workforce development into a cohesive framework anchored on four overarching and overlapping goals: (1) Improve access to cognitive neurology clinical care for older adult San Franciscans with cognitive impairment living in the most underserved and vulnerable neighborhoods of San Francisco; (2) Promote brain health literacy among older adult San Franciscans from underserved and vulnerable neighborhoods by engaging with them with educational activities centered on brain health, NCDs prevention strategies, early signs and symptoms of NCDs, and the importance of inclusive participation in NCDs clinical research studies; (3) Increase the recruitment of San Franciscans living in underrepresented and vulnerable neighborhoods into research studies underway at the UCSF Fein MAC; and (4) Conduct research focused on investigating associations between social, environmental, linguistic, and other non-biological factors and NCDs detection, diagnosis, and care among vulnerable, underrepresented San Franciscans.

### 3.4 Pillars of action

The Fein MAC COP works toward these goals through four principal community-based pillars of action that target San Franciscans living in the city’s most vulnerable and underrepresented neighborhoods. Our work is co-developed and led by our multilingual, multicultural, and multidisciplinary team of Fein MAC clinician scientists, research coordinators, and Fellows from the UCSF GBHI, working in close partnership with our community stakeholders through spontaneous communications and iterative improvements in our design and development of our programming, and through structured annual Community Advisory Boards with stakeholders to gather critical feedback to guide future plans. Figure 4 summarizes key metrics for 2025.

**Figure 4:**
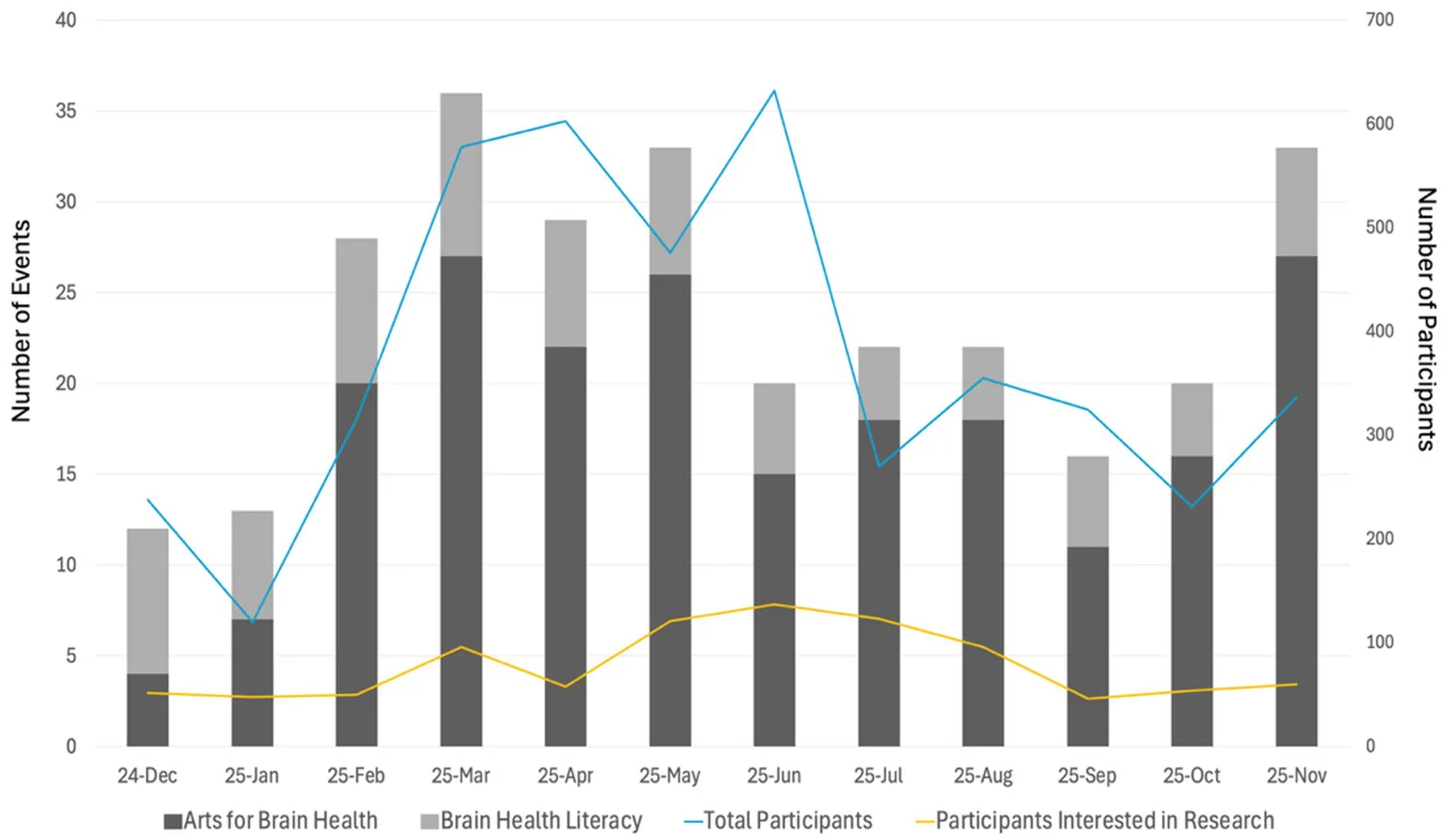
Monthly outreach events, participant attendance, and research interest generated through UCSF Fein MAC COP community engagement activities between December 2024 and November 2025. Stacked bars show the number of Arts for Brain Health (dark gray) and Brain Health Literacy (light gray) events conducted each month (left *y*-axis). The blue line indicates the total number of event participants, and the gold line indicates the number of participants who expressed interest in research participation (right *y*-axis). Values are presented by month to illustrate temporal trends in outreach activity, community engagement, and research recruitment interest.

#### 3.4.1 Brain health literacy efforts

We offer weekly to monthly educational engagements led by physicians, GBHI Fellows, and trained staff, organized in close collaboration with our community partners. We deliver our educational content in Mandarin, Cantonese, English, Tagalog, and Spanish. We offer single lectures and structured, multi-week educational programs on the brain that cover everything from basic concepts on how the brain works, how the brain changes structurally and functionally as part of normal aging, basic concepts on neuropathology, early symptoms of NCDs (including atypical presentations) and the importance of early diagnosis, evidence-based strategies to prevent cognitive decline, and the influence of social and environmental factors on brain health in San Francisco. These educational offerings run year-round, reaching thousands of participants. Importantly, brain health lectures are treated as low-barrier entry points rather than stand-alone interventions expected to produce durable change after a single exposure. Their potential value includes initiating conversations about cognitive health, building familiarity with the program, and connecting participants with repeated education, community partners, clinical services, and research opportunities.

#### 3.4.2 Arts for brain health

Beyond traditional lecture-based outreach, our team implements longitudinal arts- and activity-based initiatives in Mandarin, Cantonese, English, and Spanish to promote brain health, targeting older adults in San Francisco’s most vulnerable neighborhoods. Our flagship program, Creative Minds, funded by the San Francisco Department of Public Health, addresses key dementia risk factors, including social isolation, sedentary behavior, and limited cognitive stimulation, by fostering social connection and creative engagement through workshops in photography, mixed arts, calligraphy, creative movement, music, storytelling, and other hands-on activities. These programs also function as a bridge to underserved populations by building trust and community, enabling informal, physician-led discussions on brain health, clinical services, and research opportunities, and facilitating recruitment beyond conventional education-based outreach channels. Once a year, we host a large art exhibition showcasing the works of senior artists across neighborhoods, and we use this opportunity to offer brain health education activities in multiple languages (Supplementary Figure 1).

#### 3.4.3 Community-based clinical care

The UCSF ADRC established its first community-based clinic over 15 years ago in partnership with the Chinatown Public Health Center, serving primarily Cantonese- and Mandarin-speaking older adults. Building on this model and informed by analyses of neighborhood representation in Fein MAC research, the Fein MAC COP expanded a network of community-based clinics in collaboration with the Mission Neighborhood Health Center and the San Francisco Health Network, the city’s safety-net system. This network extends cognitive health services to underserved older adults across linguistically and socioeconomically diverse communities, including homeless populations, in partnership with four community-based primary care clinics. Collectively, these Fein MAC COP clinics provide accessible cognitive screening, diagnostic evaluation, and culturally tailored education for individuals who are uninsured, undocumented, or otherwise marginalized, with referral pathways to the Fein MAC Clinic for specialized diagnostics and advanced therapeutic and research options.

#### 3.4.4 Media engagements

The Fein MAC COP maintains a multilingual media strategy that leverages radio, television, and digital platforms to expand the reach of our brain health literacy program across San Francisco’s linguistically diverse communities. Radio and other media segments are delivered in Mandarin, Cantonese, Spanish, and English through partnerships with community media outlets that serve Chinese and Latino audiences, including local stations in Chinatown and the Mission District. These broadcasts feature culturally resonant discussions on aging, memory, and cognitive health, led by Fein MAC clinicians and community health advocates. In addition to traditional media, the program maintains a strong digital footprint, with multilingual Facebook, YouTube, and WeChat channels. Brain health videos, public service announcements, and multilingual collaborations, such as multilingual brain donation campaigns, further extend the Fein MAC COP’s visibility. These sustained media efforts aim to normalize conversations about brain health in communities that have long been underserved, effectively helping bridge the gap between academic medicine and public awareness.

## 4 Measuring impact

Through these pillars of action, we have worked to improve brain health literacy, promote successful cognitive aging, and improve early recognition of NCDs among vulnerable older adults in San Francisco. Concurrently, our work has led to a steady increase in recruitment of new research participants from nearly all of San Francisco’s diverse communities, with the most significant recruitment coming from vulnerable and underrepresented neighborhoods, including neighborhoods we had not previously reached (Figure 3; Table 1). The largest increases in new participant enrollment were observed in vulnerable neighborhoods including Bayview Hunters Point (+15 participants), Excelsior (+14), Mission (+13), and Outer Mission (+9). These gains were concentrated in neighborhoods that have historically been underrepresented in the UCSF ADRC.

**Table 1:** Changes in new UCSF ADRC participant enrollment across San Francisco neighborhoods before and after incorporation of neighborhood-based community outreach activities.

Neighborhood	Enrollment count 2010–2017	Enrollment count 2018–2025	Net change	Rate ratio (95% CI)	*p* value	Socioeconomic vulnerability rank
Bayview Hunters Point	5	20	+15	4.00 (1.46–13.64)	**0.004**	44
Excelsior	6	20	+14	3.33 (1.29–10.14)	**0.009**	27
Mission	11	24	+13	2.18 (1.03–4.93)	**0.041**	27
Outer Mission	3	12	+9	4.00 (1.08–22.09)	**0.035**	31
Oceanview/Merced/Ingleside	2	8	+6	4.00 (0.80–38.67)	0.109	36
Bernal Heights	4	10	+6	2.50 (0.72–10.92)	0.180	31
Tenderloin	3	8	+5	2.67 (0.64–15.61)	0.227	25
North Beach	3	7	+4	2.33 (0.53–13.98)	0.344	36
Nob Hill	1	4	+3	4.00 (0.40–196.99)	0.375	41
Portola	2	4	+2	2.00 (0.29–22.11)	0.688	22
Inner Richmond	5	7	+2	1.40 (0.38–5.59)	0.774	20
Lakeshore	1	2	+1	2.00 (0.10–117.99)	1.000	40
Glen Park	4	5	+1	1.25 (0.27–6.30)	1.000	9
West of Twin Peaks	15	16	+1	1.07 (0.49–2.32)	1.000	7
Presidio	0	1	+1	Inf (0.03-Inf)	1.000	10
Chinatown	2	2	+0	1.00 (0.07–13.80)	1.000	44
Mission Bay	1	1	+0	1.00 (0.01–78.50)	1.000	19
Pacific Heights	8	8	+0	1.00 (0.33–3.06)	1.000	5
Russian Hill	7	7	+0	1.00 (0.30–3.34)	1.000	15
Seacliff	1	1	+0	1.00 (0.01–78.50)	1.000	29
Sunset/Parkside	15	15	+0	1.00 (0.46–2.20)	1.000	41
Treasure Island	1	1	+0	1.00 (0.01–78.50)	1.000	4
Western Addition	8	6	−2	0.75 (0.21–2.46)	0.791	38
Noe Valley	7	5	-2	0.71 (0.18–2.61)	0.774	2
Potrero Hill	5	3	-2	0.60 (0.09–3.08)	0.727	13
Twin Peaks	4	2	-2	0.50 (0.05–3.49)	0.688	35
Hayes Valley	5	2	−3	0.40 (0.04–2.44)	0.453	16
Japantown	4	1	−3	0.25 (0.01–2.53)	0.375	23
Visitacion Valley	4	1	−3	0.25 (0.01–2.53)	0.375	43
Outer Richmond	11	7	−4	0.64 (0.21–1.80)	0.481	11
Inner Sunset	10	6	−4	0.60 (0.18–1.82)	0.454	1
South of Market	10	6	−4	0.60 (0.18–1.82)	0.454	25
Presidio Heights	7	3	−4	0.43 (0.07–1.88)	0.344	2
Financial District/South Beach	9	4	−5	0.44 (0.10–1.59)	0.267	34
Marina	8	3	−5	0.38 (0.06–1.56)	0.227	18
Haight Ashbury	9	3	−6	0.33 (0.06–1.34)	0.146	8
Lone Mountain/USF	9	3	−6	0.33 (0.06–1.34)	0.146	24
Castro/Upper Market	14	2	−12	0.14 (0.02–0.62)	**0.004**	17

Neighborhood-specific counts of newly enrolled participants during two equal eight-year periods. For each neighborhood, the table presents the net change, later-versus-earlier enrollment rate ratio with an exact 95% confidence interval, two-sided exact *p*-value, and overall neighborhood vulnerability rank. Vulnerability ranks were derived using the same composite rank-sum method presented in Figure 2 and range from 1 (least vulnerable) to 45 (most vulnerable). Neighborhoods are ordered by net change in enrollment. Positive values indicate higher enrollment during 2018–2025, whereas negative values indicate lower enrollment. Bolded values indicate statistical significance at *p* < 0.05.

To ensure that research participants from vulnerable backgrounds benefit as much as possible from their participation, they are offered financial compensation for their time (up to $300 per research visit) and transportation and food vouchers. Moreover, our teams prepare research visit summaries for each participant, which include all clinically relevant information gathered during the visit. Additionally, the Fein MAC COP team organizes annual research events for active research participants and their families, sharing the main research findings and recommendations with the broader communities served. In this way, we build on our mission of conducting research with and for older adults in San Francisco.

The activities of the Fein MAC COP have catalyzed meaningful advances in research investigating brain health disparities. Through cross-sector partnerships, the program has generated data and collaborations that bridge social epidemiology and translational neuroscience. By aligning outreach with social determinants of brain health, the Fein MAC COP has built research cohorts that mirror the diversity of the Bay Area, not just by race and ethnicity, but also by language, culture, and neighborhood-specific exposures. These efforts have also led to innovative studies exploring cognitive health among Bay Area populations. Through cross-sector partnerships, the Fein MAC COP has supported studies of brain health among populations historically underrepresented in aging research, including homeless Latino adults, Chinese older adults, and multilingual communities. These collaborations have contributed to multilingual assessment tools, culturally and linguistically adapted recruitment and evaluation, and research examining how social, environmental, linguistic, genetic, and lifestyle factors shape cognitive aging. Community-based clinics and culturally resonant arts and media activities have also created pathways into clinical services and research for residents who might otherwise remain beyond the reach of academic programs. These combined activities have transformed the Fein MAC’s outreach efforts from a largely unidirectional educational endeavor into an evidence-generating, participatory framework that promises to inform the biological, psychosocial, and environmental dimensions of late-life brain health disparities.

## 5 Discussion

The Community Outreach Program (COP) of the UCSF Fein MAC proposes a pragmatic, data-driven approach to community outreach, recruitment, and engagement that aligns public health principles with research on the root causes of age-related neurocognitive disorders (NCDs) and other brain health disparities. One of the program’s strengths lies in its place-based, social and environmental determinants-oriented framework, which grounds outreach and research in the social and environmental realities of San Francisco’s neighborhoods and the residents’ lived experiences. By focusing on the structural and contextual factors that shape brain health across racial and ethnic groups, the program seeks to achieve greater precision in identifying populations at elevated risk for age-related neurocognitive disorders (NCDs). We believe this approach can complement traditional race- or ethnicity-based outreach, recruitment, and engagement frameworks.

A few key lessons have emerged from implementing this neighborhood-based approach. We have summarized these in Supplementary Table 2 and discuss four main lessons here. First, we believe that by emphasizing race- and ethnicity-associated disparities rather than attributing these disparities to race or ethnicity itself, we minimize the risk of reifying racial and ethnic brain health disparities as biologically caused and instead contribute to situating population health differences within their local social, historical, and environmental contexts. This orientation mitigates key risks we believe are inherent to more traditional race and/or ethnicity-based outreach and engagement models: anchoring disparities in presumed, immutable, intrinsic racial and/or ethnic characteristics; homogenizing within-group diversity; overlooking multiracial/multiethnic groups; and failing to recognize vulnerable subgroups within historically privileged groups.

Second, implementation of the Fein MAC COP has shown that individual outreach events and sustained community engagement serve different but complementary purposes. A lecture, health fair participation, media appearance, or arts event can provide a relatively low-barrier introduction to brain health information and to the academic center, but a single encounter should not be assumed to produce durable changes in knowledge, trust, healthcare use, or research participation. Within the COP, these activities are therefore used as entry points into a broader engagement pathway that includes repeated educational contact, referrals from trusted community partners, community-based clinical services, research opportunities, and the return of information and other benefits to participants. This longitudinal approach requires substantially more coordination, staff continuity, and institutional commitment than isolated outreach events. It also creates practical challenges: relationships take years to develop, communities reasonably expect continued presence after a grant or project ends, and short-term funding mechanisms rarely support the infrastructure needed to maintain trust. The COP has addressed these challenges through continuing ADRC support, collaboration across projects with overlapping goals, philanthropic support, volunteer contributions, and partnerships with organizations that possess community credibility. These arrangements, however, remain resource-intensive and do not fully resolve the program’s sustainability needs.

Reciprocity is central to this model rather than an ancillary benefit. In practice, reciprocity has included compensating research participants, providing transportation and food, returning clinically relevant research-visit summaries in accessible formats, discussing clinically-relevant findings with participants and families, supporting multilingual brain health education, co-developing activities with community partners, and creating community-facing programs such as arts exhibitions. For centers seeking to adapt this approach, the transferable lesson is not that every COP activity must be reproduced. Rather, institutions should identify locally trusted partners, define what they can reliably contribute over time, incorporate community benefit and participant support into program planning, and evaluate outreach activities both for their immediate purpose and for their role in creating pathways to deeper engagement.

Third, we believe that our place-based approach to community outreach and engagement offers a pragmatic, data-driven operational framework and methodology for any academic center engaged in studying and mitigating brain health disparities locally, including those situated in regions with relatively little racial or ethnic diversity, yet substantial differences in age-related brain health outcomes, access to diagnosis and care, and other disparities remain. Research shows that, in largely homogeneous racial and/or ethnic settings, these disparities are associated with region-specific structural factors. For instance, in a predominantly White U. S. cohort of 388 middle- to older-aged adults (88% non-Hispanic White), a higher Area Deprivation Index (ADI, a composite of socioeconomic vulnerability per U. S. census block group) was associated with greater white matter hyperintensity burden on MRI after adjustment for age, sex, education, smoking, and visit timing (32). Similarly, in a larger and racially homogeneous (mostly White participants) UK Biobank sample of 19,638 adults, higher neighborhood deprivation, indexed by the Townsend deprivation scale (based on UK census socioeconomic data), was associated with smaller hippocampal volume, greater white matter hyperintensity burden, poorer cognition, and thinner cortex in multiple brain regions.^33^ Similar studies conducted in racially and ethnically diverse U. S. populations show that greater regional disadvantage is independently associated with lower total cerebral and hippocampal volumes after adjustment for race and ethnicity (18), further supporting the overarching hypothesis driving our neighborhood-based approach.

Residential segregation provides an important structural context for the COP’s neighborhood-based approach, but racial or ethnic clustering is not a prerequisite for the model. Historical and contemporary housing discrimination, displacement, unequal investment, environmental burden, and differential access to healthcare and other resources have contributed to geographic variation in conditions relevant to brain health. The COP therefore uses neighborhoods not as proxies for racial or ethnic populations, but as units through which accumulated social and environmental vulnerability can be identified and addressed. This distinction is especially important in San Francisco, where socioeconomically vulnerable neighborhoods include residents from multiple racial and ethnic groups and where members of the same racial or ethnic population may live in neighborhoods with markedly different resources, exposures, and health risks.

Fourth, our work suggests that a place-based approach to community outreach, recruitment, and engagement provides a more generalizable and analytically robust framework for understanding brain health vulnerability than race- and/or ethnicity-based approaches. The wide availability of validated region-specific vulnerability indices, such as the ADI, greatly facilitates the implementation of this outreach approach by enabling academic centers to identify and prioritize communities using empirically derived measures of neighborhood disadvantage. By leveraging such tools, academic efforts can be more precisely targeted, transparent, and evidence-based, aligning resources with demonstrable need. Still, we recognize that place-based outreach may be less effective when the defining characteristic of the intended population is geographically dispersed; for example, a particular language or dialect, ethnic or cultural affiliation, rare clinical condition, migration history, or social network. In such circumstances, geographic targeting should be complemented by language-, culture-, clinic-, organization-, network-, or condition-based outreach. Thus, the neighborhood-based framework is not proposed as a universal replacement for race-, ethnicity-, or culture-informed engagement, but as a means of aligning outreach more directly with locally measured conditions that shape brain health and research participation.

A persistent challenge for the Fein MAC COP is sustaining funding for the longitudinal, relationship-based work required for effective community engagement. Although the National Institutes of Health prioritizes hypothesis-driven research, its funding mechanisms are less suited to supporting the long-term infrastructure and sustained community presence necessary for outreach and trust-building. Meaningful partnerships, clinical integration, and community credibility require years to develop and are difficult to maintain within short funding cycles. While the UCSF ADRC provides essential, continuous support for core outreach and recruitment activities, its resources are insufficient to sustain the full scope of the COP. We have partially addressed this gap by aligning with other NIH-funded Fein MAC projects with overlapping goals; however, a substantial portion of COP activities remains dependent on philanthropy and volunteerism. This reflects a broader structural challenge in academic medicine: programs essential to representative research participation and translation often lack stable funding despite their central role in advancing public health.

In conclusion, this report is the first paper to describe the Fein MAC COP as an integrated program and is intended to introduce its neighborhood-based framework, partnerships, and pillars. The present data describe program reach and enrollment patterns but do not permit formal comparisons of the effectiveness, cost, or cost-effectiveness of individual components. Prospective process, resource, and outcome measures are being developed, and separate manuscripts will describe individual pillars in greater implementation and evaluative detail. Accordingly, looking ahead, the Fein MAC COP aims to further integrate its social and environmental determinants of brain health framework across clinical and research endeavors. Central to this effort is the development of longitudinal, multilevel datasets that link neighborhood- and participant-level exposures to blood-based biomarkers, neuroimaging, and cognitive trajectories, advancing a biosocial model of brain aging. Additional priorities include expanding partnerships with arts organizations, media platforms, and safety-net healthcare systems, as well as strengthening evaluation frameworks to capture the impact of outreach on research participation, representation, community empowerment, social connection, well-being, and trust. Ultimately, the goal is to establish a scalable and replicable model that academic centers can adapt to address neurocognitive health disparities through integrated community engagement and research.

## Data Availability

Publicly available datasets were analyzed in this study. This data can be found: National Alzheimer’s Coordinating Center (https://www.naccdata.org/).
