# Supplementary material for: From neighborhoods to brain health: an outreach model linking health literacy, cognitive care, research, and the arts, in San Francisco
Source: Front Public Health. 2026 Sep 17;14:1930344. doi: 10.3389/fpubh.2026.1930344 (PMC13626907; doi:10.3389/fpubh.2026.1930344)
Supplement: Supplementary file 1 [file Table_1.DOCX]

**Supplementary Table 1:** **UCSF Fein MAC COP San Francisco Partners and Collaborators.** List of partners and collaborators, organized by neighborhood

| **San Francisco Neighborhood** | **Partners Located in Neighborhood** |
| --- | --- |
| Alamo Square | 3rd Street Baptist Church |
| Bayview Hunters Point | Bayview Adult Day Health Center; Dr. George W. Davis Senior Center; Network for Elders; Project Wreckless; Rafiki Coalition; San Francisco Black/African American Faith Based Coalition; Southeast Health Center; YMCA Bayview Hunters Point |
| Bernal Heights | YMCA Mission Branch |
| Civic Center | Consulate of El Salvador |
| Downtown/Financial District | Philippine Consulate of San Francisco |
| Fillmore District / Western Addition | National Council of Negro Women- Golden Gate Section |
| Financial District | Consulate of Colombia; Consulate of Guatemala; Family Caregiver Alliance; Leukemia & Lymphoma Society- Northern California Blood Cancer Conference; Self-Help for the Elderly |
| Glen Park | On Lok 30th Street Senior Center |
| Hayes Valley | San Francisco Conservatory of Music |
| Ingleside Heights | IT Bookman Community Center |
| Inner Richmond | Links Inc. - San Francisco Chapter; SF Village, |
| Inner Sunset | Cayuga/Community Living Campaign (At St. Anne's Church) |
| Lower Pacific Heights | Booker T Washington Community Service Center; Sequoia Living (The Sequoias) |
| Mission | Asian Pacific Islander Legal Outreach; Centro Latino of San Francisco; Community Music Center; Good Samaritans Family Resource Center; Latino Community Partnership; Living City Sunday Streets; Mission Cultural Center for Latino Arts; Mission Neighborhood Center Inspiring Success; Mission Neighborhood Health Center; Mission Neighborhood Resource Center; Mission San Francisco Public Library; Pilipino Senior Resource Center; Ruth's Table; San Francisco General Hospital |
| Mission Bay | UCSF Dream Lab; UCSF Filipino American Medical Student Association; UCSF Helen Diller Family Comprehensive Cancer Center |
| Mission Dolores | Mission Housing Development Corporation |
| Mission Terrace | Clinic by the Bay (Bethel Center) |
| Nob Hill | Chinatown Public Health Center; Chinese Hospital |
| North Beach | Telegraph Hill Neighborhood Center |
| Outer Richmond | National Alliance on Mental Illness (NAMI)- SF |
| Richmond | OnLok PACE |
| SOMA | 106 KMEL Radio; Canon Kip Senior Center; Filipino Community Development Corporation; Hecho en California Radio Program; Mabuhay Health Center; Medical Respite & Sobering Center; SOMA PILIPINAS; SOMArts Cultural Center; SOMCAN |
| South Beach | Consulate of Mexico, Ventanilla de Salud |
| Tenderloin | SF Recovery Theatre |
| Union Square | Consulate of Peru |
| Western Addition | UCSF Black Wellness Center |

**Supplementary Table 2. Key Lessons from the Fein MAC COP and Implications for Adaptation in Other Academic Settings**

| **Key lesson** | **Fein MAC COP experience** | **Transferable guidance** | **Tradeoff or limitation** |
| --- | --- | --- | --- |
| Target place-based vulnerability, not racial or ethnic proxies | San Francisco neighborhoods with socioeconomic vulnerability include residents from multiple racial and ethnic groups, while members of the same racial or ethnic group experience different neighborhood conditions. | Use locally validated geographic indicators to prioritize need while retaining race-, ethnicity-, language-, and culture-informed strategies when relevant. | Area-level measures can conceal vulnerable individuals within advantaged areas and should not be treated as individual-level attributes. |
| Build relationships before research recruitment is needed | Repeated work with community organizations, leaders, clinics, cultural institutions, and residents created access and credibility that could not be generated at the moment recruitment began. | Establish trusted partners, shared expectations, and communication channels before launching a study-specific campaign. | Requires staff continuity and institutional presence; relationships can be harmed if engagement ends when a grant closes. |
| Use one-time events as gateways, not complete interventions | Lectures, fairs, media appearances, and arts events provide low-barrier introductions but are embedded in pathways to repeated education, referrals, clinical services, and research. | Evaluate events for immediate reach and for subsequent engagement, referrals, return attendance, research interest, or clinical follow-up. | A single encounter should not be assumed to produce durable changes in knowledge, trust, or behavior. |
| Make reciprocity concrete | The COP compensates participants, supports transportation and food needs, returns visit summaries and research findings, and offers community-facing education and arts programming. | Define and budget what participants and partners receive in return for their time, knowledge, and trust. | Reciprocity is resource-intensive and must reflect priorities identified with communities rather than benefits defined only by the institution. |
| Combine outreach modalities | Community partners, clinics, multilingual education, arts, radio, television, and other activities reach residents through different trusted settings. | Use complementary interpersonal, clinical, cultural, and media channels rather than assuming one method reaches every population. | The most effective mix is context-specific; the present Community Case Study cannot rank components comparatively. |
| Integrate the pillars | Education, clinical services, community arts, and other activities can be organized into pillars of action and provide multiple entry points that reinforce one another. | Track participant pathways across pillars rather than evaluating every activity only as an isolated event. | Requires shared coordination, referral processes, data definitions, and privacy-conscious tracking. |
| Plan for long-term sustainability | Core UCSF ADRC support is supplemented by aligned grants, philanthropy, and volunteer contributions, yet the full relationship-based infrastructure remains difficult to sustain. | Separate start-up, event-specific, and ongoing infrastructure needs; diversify funding and protect core relationship-building roles. | Short funding cycles are poorly aligned with the years required to build trust and create expectations of continued presence. |
| Adapt the model to the target and setting | Place-based targeting is useful where brain health risks or access barriers vary geographically; dispersed language, cultural, clinical, or network-defined populations may require other strategies. | Preserve the principles of local data, partnership, reciprocity, multimodal engagement, and continuous learning while changing the geographic unit or outreach channel. | Neighborhood outreach is complementary, not universally superior; the correct unit depends on the outreach objective. |

**Note.** Fein MAC, Fein Memory and Aging Center; COP, Community Outreach Program. These lessons reflect an evolving community case study and are considerations for local adaptation, not evidence of the comparative effectiveness or cost-effectiveness of individual components. Component-level evaluation will require prospective measures of reach, dose, participant pathways, resources, implementation fidelity, and outcomes.

Supplementary figure 1: 2025 Creative Minds Community Arts Exhibition Flyer. Example of annual Creative Minds program community arts exhibition, hosted by UCSF Fein MAC COP. This annual exhibition brings together all senior community artists and their creations completed during the year. Artists are encouraged to bring their families and friends. Community partners are also invited. Multilingual brain health education is incorporated into the exhibition schedule.
